# Selective Targeting of Cancer Stem Cells (CSCs) Based on Photodynamic Therapy (PDT) Penetration Depth Inhibits Colon Polyp Formation in Mice

**DOI:** 10.3390/cancers12010203

**Published:** 2020-01-14

**Authors:** Jun Ki Kim, Mi Ran Byun, Chi Hoon Maeng, Yi Rang Kim, Jin Woo Choi

**Affiliations:** 1 Biomedical Engineering Research Center, Asan Institute for Life Sciences, Asan Medical Center, Seoul 05505, Korea; kim@amc.seoul.kr; 2 Department of Convergence Medicine, University of Ulsan College of Medicine, Seoul 05505, Korea; 3 Department of Pharmacology, College of Pharmacy, Kyung Hee University, Seoul 02447, Korea; bml1230@naver.com; 4 Department of Hemato-Oncology, College of Medicine, Kyung Hee University, Seoul 02447, Korea; mchihoon@khu.ac.kr; 5 Department of Hemato-Oncology, Yuseong Sun Hospital, Daejeon 34084, Korea; 6 Oncocross Ltd., Seoul 04168, Korea

**Keywords:** cancer stem cells, green fluorescent protein, colon cancer, photodynamic therapy, rose bengal photosensitizers

## Abstract

Targeting cancer stem cells (CSCs) without damaging normal stem cells could contribute to the development of novel radical cancer therapies. Cells expressing leucine-rich repeat-containing G-protein coupled receptor 5 (Lgr5) constitute a cancer-causing population in the colon; therefore, targeting of Lgr5+ cells is expected to provide an opportunity to mitigate colon cancer. However, the expression of Lgr5 in normal stem cells makes it difficult to prove the efficacy of therapies targeted exclusively at Lgr5+ cancer cells. We used a modified photodynamic therapy technique involving cellular radiative transfer between green fluorescent protein (GFP)-expressing cells and a rose bengal photosensitizer. After treatment, tumors containing GFP-Lgr5+ cells were observed to be significantly suppressed or retarded with little effect on GFP-Lgr5+ stem cells at the crypt bottom. Lgr5+ CSCs were specifically eradicated in situ, when localized based on the depth from the colon lumen, revealing the potential preventive efficacy of Lgr5-targeted therapy on tumor growth. This study supports the idea that Lgr5+ cells localized near the colon luminal surface are central to colorectal cancer. With further development, the targeting of localized Lgr5+ cancer stem cells, which this study demonstrates in concept, may be feasible for prevention of colon cancer in high-risk populations.

## 1. Introduction

Cancer stem cells (CSCs) are a malignant population of cancer cells with stem-cell-like properties that help develop and maintain cancer [1]. Although the existence of CSCs was initially controversial, the role of CSCs was since demonstrated in a variety of cancers, including lung cancer, brain tumors, and cancer of the head and neck [2,3,4,5]. CSCs play a crucial role not only in the generation of primary cancer, but also in metastasis [1,6]. In addition, since CSCs are associated with resistance to chemotherapeutic drugs [6], targeting CSCs and their markers selectively is a significant step to improving the effectiveness of cancer therapies [7].

Among the CSCs, the leucine-rich repeat-containing G-protein coupled receptor 5-positive (Lgr5+) stem cells are known to act as the “seed” of colonic adenoma and cancer [8,9]. While Lgr5+ cells of normal crypts or colonic polyps which are non-precancerous lesions are located only at the bottom of the crypt, Lgr5+ cells of colon cancers and precancerous lesions like colonic adenomas are observed at all portions of glands in human cancer samples [10]. In addition, formation of adenoma in the colon was shown to result from a single mutation in the Lgr5+ cells at the crypt bottom layer, whereas the same mutation in the epithelial cells above the bottom layer does not result in lesion development [8]. In normal intestinal cells, Lgr5 expression is an exclusive marker for stemness [11,12], while, in colorectal cancer, Lgr5+ cells play an important role in carcinogenesis, but are localized to the luminal surface during early cancer development [8,13,14]. Selective ablation of Lgr5+ cells induces tumor regression in organoids, while a re-emergence of Lgr5+ cells was observed to drive tumor re-growth [15]. Thus, targeted therapy for Lgr5+ cells in colon cancer might prevent the formation of colon adenoma or inhibit the progression of early-stage colon cancer. Furthermore, Lgr5+ cells are an attractive treatment target in terms of drug development, since the structure of Lgr5 was fully determined and Lgr5 is localized on the cellular surface. This was recently demonstrated via antibody–drug conjugates targeting Lgr5+ cells, which resulted in a reduction in tumor size, extended patient survival, and prevention of cancer recurrence [16,17]. However, since Lgr5 is expressed in CSCs, as well as normal stem cells, non-selective therapies targeting Lgr5 may lead to significant damage of noncancerous (normal) colon stem cells and destruction of normal colon homeostasis. For these reasons, non-selective Lgr5-targeting therapies are difficult to apply in clinical settings.

It was previously reported that Lgr5+ colon stem cells move from the crypt to the luminal epithelium following the administration of a carcinogen, and that Lgr5-dense foci grow faster than Lgr5-sparse foci [18]. The difference in location between normal colon stem cells, which congregate at the crypt bottom, and colon cancer stem cells, which migrate toward the luminal epithelium [14], offers a potential means of targeted preventative treatment when combined with photodynamic therapy (PDT).

Although PDT methods are utilized to treat colorectal cancer stem cells [19], a classic limitation of PDT is that the incident light intensity drops off as a function of tissue depth. When combined with a novel fractionated PDT method for sensitizing and killing green fluorescent protein (GFP)-expressing cells [20,21], it becomes possible to focus treatment on colon CSCs at the luminal surface, while minimizing damage to normal stem cells. Using this minimally invasive fractionated PDT technique, we further reveal the role of Lgr5+ CSCs in tumor maintenance and proliferation, demonstrating the therapeutic potential of localized PDT for targeting Lgr5+ cells in sporadic tumor models while minimizing damage to off-target tissues. While the current treatment model requires CSCs to express GFP, with development of conjugated Lgr5-targeting photosensitizers, the localization method demonstrated here has the potential to allow targeted early and preventative treatment for patients at high risk of hereditary colorectal cancer.

## 2. Materials and Methods

### 2.1. Photodynamic Therapy of Mouse Colon Adenocarcinoma

To ensure uniform delivery of laser radiation onto the colon cancer cells, custom-made cylindrical diffuse fibers were used (SOMTA, Ltd., Pietermaritzburg, South Africa). The light propagated through the optical fiber core was emitted by a diffusing part, from which light disperses in a radially uniform manner. The cylindrical diffuse fiber is bendable, and the diameter and length of the diffuser were 600 µm and 2 cm, respectively. After gently inserting the diffusing fiber into the mouse colon via the anus, blue light (473 nm wavelength) from a diode laser was delivered through the optical waveguide for uniform irradiation. The radiation power of the diffusing part per unit area was adjusted to fall between 22 mW/mm^2^ and 25 mW/mm^2^. Fiber transmission loss was negligible because of the short length of the non-diffusive fiber used (5 m long).

### 2.2. Polyp Growth and Treatment in GFP-Lgr5 Mice

Wild-type and Lgr5-EGFP-IRES-creERT2 knock-in mice (GFP-Lgr5 mice) were randomized into three groups each (*n* = 5 per group), with a total of six groups: a wild-type negative control, a GFP-Lgr5+ negative control, and four groups with sporadic tumors, half of which were treated by PDT and half of which were left as a positive control. The azoxymethane (AOM)/dextran sulfate sodium (DSS) mouse method [22,23] was used to generate inflammatory sporadic precancerous lesions and tumors. Azoxymethane (AOM) (10 mg/kg body weight) was intraperitoneally injected into the treatment and positive control groups for wild type and GFP-Lgr5 mice. One week after the injection, the mice were administered 2% dextran sulfate sodium (DSS) in drinking water for seven days, followed by DSS-free water for another seven days. This on/off DSS administration cycle was repeated three times. Wild-type and GFP-Lgr5 mice in the treatment group were intravenously injected with rose bengal (RB) (50 nM, 0.75 mL/kg), a photosensitizer which spreads to the colon through the vasculature, followed by 2 min of blue light (473 nm) radiation through anal insertion 4 h after the RB injection, twice a week for seven weeks.

All animal experiments were performed according to protocols approved by the Institutional Animal Care and Use Committee (IACUC) of Kyung Hee University (KHUASP(SE)-17-048-1). The committee also followed the guidelines set by the Institute of Laboratory Animal Resources (ILAR), following the Laboratory Animal Act of the Republic of Korea.

### 2.3. In Vivo Imaging of the Polyps in GFP-Lgr5 Mice

Polyp growth was monitored by endoscopic measurement of the polyp diameter. After anesthesia induction, mice were placed on a stage for colonoscopy. An endoscope comprising a rigid, straight telescope (ColoView; Karl Storz, Inc., Tuttlingen, Germany) was used in combination with a tunable xenon lamp (XENON nova 175; Karl Storz, Inc., Tuttlingen, Germany). This colonoscope (outer diameter 1.9 mm) was introduced through the anus, while the colon was carefully insufflated using an air pump to avoid colon wall collapse and to secure a clear view. Videos were acquired using a three-chip camera with high imaging quality and recorded on a computer. Individual images were captured from the recorded video files by using frame-extracting software. Tumor sizes were estimated from the images (width × height × 2). The distance-dependent magnification of the colonoscope was calibrated by imaging a ruler in the same field. Observed tumor coordinates were read from a guide on the colonoscope head and used to select an appropriate depth for diffuse fiber insertion.

To compare the tumor size before and after PDT, well-isolated tumors were selected, and the coordinates of the colonoscope were recorded in detail for each tumor. The tumor location was determined based on previously measured coordinates. To calibrate the size of the tumor, after focusing on a tumor, the image of a ruler was captured in the same focal plane.

### 2.4. Visualization of In Vivo Cell Death

To evaluate cell death in live animals, the fluorescent red FLIVO™ caspase activity probe (emission = 550–580 nm, excitation = 590–600 nm, Immunochemistry Technologies LLC, AbCys SA, Paris, France) was diluted in phosphate-buffered saline (PBS) containing 1% dimethyl sulfoxide (DMSO) at a dose determined by the body weight of the animal, and then intravenously injected into wild-type and GFP-Lgr5 mice. The mice were sacrificed 1 h later, and their colons were isolated. The fluorescence signal from the FLIVO™ probe was captured using a conventional laser scanning confocal microscope.

### 2.5. Histological and Gene Expression Analyses

The histological analysis of epithelial denudation, mast-cell infiltration, tissue fibrosis, and apoptosis were done by immunostaining with cytokeratin, toluidine blue staining (8544-4125; Daejung Chemicals & Metals, Seoul, Korea), Masson’s trichrome staining (Junsei Chemical, Tokyo, Japan), and TUNEL (Terminal deoxynucleotidyl transferase dUTP nick end labeling) staining (1 684 795; Roche, Mannheim, Germany), respectively, as previously described [2,3,4].

### 2.6. In Vitro Treatment Power Calibration on Cells

Human Lung cancer H460 and colorectal cancer DLD-1 cells were cultured in RPMI 1640 medium (Roswell Park Memorial Institute medium, HyClone, GE Healthcare Life Sciences, Marlborough, MA, USA) containing 10% fetal bovine serum (FBS; Gibco, Thermo Fisher Scientific, Waltham, MA, USA) and antibiotics (HyClone, GE Healthcare Life Sciences). H460 and DLD-1 cell lines were authenticated by short tandem repeat profiling. Mouse melanoma B16 cells were maintained in DMEM (Dulbecco’s Modified Eagle Medium, HyClone, GE Healthcare Life Sciences) supplemented with 10% FBS and antibiotics. Stable cells for GFP expression were transfected with GFP via Lipofectamine^®^ 2000 reagent (Thermo Fisher Scientific) before being plated on culture dishes. For the cell survival assay, 100 nM rose bengal (RB) was mixed into each cell culture medium, incubated for 4 h, and irradiated with a 473-nm laser at 7 mW/cm^2^, following attenuation by 200 microns of isolated colon tissue from a wild-type mouse. The cell viability was measured 24 h later with a 3-(4,5-dimethylthiazol-2-yl)-2,5-diphenyltetrazolium bromide (MTT) assay.

### 2.7. Colon Adenocarcinoma Organoid

Lgr5-EGFP-IRES-creERT2 knock-in mice were administered 10 mg/kg AOM by intraperitoneal injection, and then subjected to three on/off cycles of 2% DSS-containing drinking water. The mice were then euthanized, and polyps were isolated. The polyps were washed with ice-cold PBS and minced. The minced tissue fragments were incubated at 37 °C in DMEM/F-12 containing 2 mg/mL collagenase II. After 2 h, the fragments were washed with PBS, incubated in 0.025% Trypsin-EDTA (HyClone, GE Healthcare Life Sciences) for 3 min, and mixed with the FBS-containing medium. This slurry solution of tissue fragments was passed through a 40-mm cell strainer to separate the single cells from the polyps and centrifuged at 300× *g* for 5 min. The cell pellets were resuspended in Matrigel (BD Matrigel™ Basement Membrane Matrix, Cat # 356230, BD Biosciences, Bergen, NJ, USA) and plated. After solidification of the Matrigel, colon organoid medium (DMEM/F12 containing 500 ng/mL R-spondin 1, 100 ng/mL Noggin, and 50 ng/mL EGF (epithelial growth factor, Pepro Tech, Rocky Hill, NJ, USA) was added. Two weeks later, the organoids were collected, trypsinized, centrifuged, and divided into two aliquots. Then, the aliquots were treated with or without 100 nM RB for 4 h and irradiated with blue light at a fluence rate of 7 mW/cm^2^. After irradiation, they were cultured for seven days, and the organoid number was counted.

### 2.8. Immunohistochemistry

Laser-irradiated mice colons were isolated, washed with ice-cold PBS, and fixed with 4% paraformaldehyde for 24 h. The fixed colons were dehydrated by consecutive incubations in 70%, 80%, 90%, 95%, and 100% ethanol for 2 h each, after which they were embedded in 100% paraffin overnight, and then solidified and sectioned at 4-μm thickness. The tissue sections were subsequently deparaffinized and rehydrated, and their endogenous peroxidase was blocked by 15 min of incubation in 3% hydrogen peroxide. Antigen retrieval was performed by boiling the sections in a solution of 10 mM sodium citrate and 0.05% Tween-20 (pH 6.0) for 15 min. Afterward, the sections were incubated with the primary antibodies overnight at 4 °C, rinsed with PBS containing 0.05% Tween-20, and incubated with the secondary antibodies (vector lab, Burlingame, CA, USA). Finally, the slides were counterstained with Mayer’s hematoxylin and mounted for microscopic analyses. The primary antibodies used were as follows: anti-Ki-67 (1:5000, Abcam, Cambridge, UK, ab15580) and anti- tumor necrosis factor (TNF)-α (1:100, Santa Cruz Biotechnology, Dallas, TX, USA, sc-133192).

### 2.9. Statistical Analysis

All the statistical tests were two-sided, and a *p*-value less than 0.05 was considered to indicate a statistically significant difference. Student’s *t*-tests were used to evaluate the statistical significance by comparing the differences between the treatment and control groups. Analyses were performed on GraphPad Prism 6.0 software.

## 3. Results

**Figure 1:**
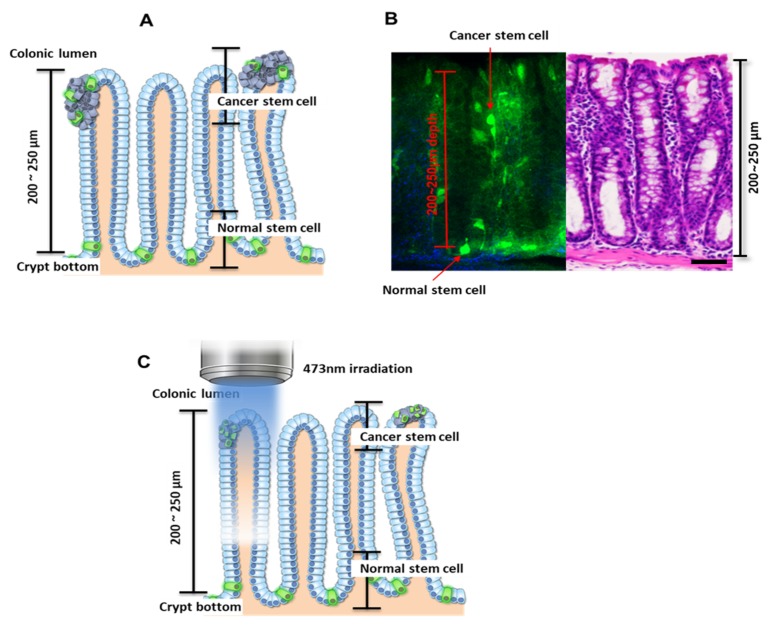
Locations of noncancerous and cancerous leucine-rich repeat-containing G-protein coupled receptor 5-positive (Lgr5+) cells in the colon. (**A**) Schematic depicting the length of the colon crypt and locations of colon stem cells. (**B**) Confocal image and H&E (Hematoxylin and Eosin) staining of the Lgr5-EGFP-IRES-creERT2 knock-in mouse model. Scale bar: 50 µm (**C**) Schematic of the novel photodynamic therapy (PDT) method applied to selectively eliminate cancer stem cells while minimizing the effects on normal stem cells at the crypt bottom. This is possible because of the limited penetration depth of the 473-nm laser and the propensity of cancer stem cells and normal stem cells to localize in different parts of the colon. Following the novel PDT method, cancer stem cells were selectively destroyed and tumor formation was decreased.

Cells expressing Lgr5 are known to localize at the crypt bottom if they are normal (noncancerous), and nearer to the colonic lumen if cancerous (Figure 1A) [9,10,14,18,24]. Consistent with previous observations, cells expressing Lgr5 were observed at depths of 200–250 µm from the top of the colon crypt in the control mice [18]. In mice treated with AOM/DSS, the Lgr5+ cells were observed to migrate to the luminal surface after AOM treatment followed by DSS administration (Figure 1B), and these were considered to be colon cancer stem cells. In addition, many Lgr5+ cells were observed inside the colonic adenoma using fluorescence microscopy (Movie S1, Supplementary Materials)).

PDT is the treatment to induce cancer cell apoptosis through the chemical reaction of oxygen and external light by administering a photosensitizer, a substance that is sensitive to light [25]. Rose bengal (4,5,6,7-tetrachloro-2′,4′,5′,7′-tetraiodofluorescein) is a well-known photosensitizer, and activated Rose bengal produces the conversion of tissue oxygen to singlet oxygen and free radicals. This ROS (reactive oxygen species) production is achieved upon irradiation by light of rose bengal, whose intense absorption wavelength ranges between 480 nm and 550 nm [26,27]. Accumulation of free radicals by PDT induces cellular damage and apoptosis. Also, PDT is well known to be dependent on the penetration depth of light into tissue. To destroy CSCs with minimal effect on normal stem cells, we utilized the penetration depth of a 473-nm laser. Since the penetration depth was less than 250 µm, the localization of normal cells deeper in the crypt bottom protected them from PDT, whereas cancer cells in the colonic lumen were exposed to light-generated reactive oxygen species (Figure 1C).

**Figure 2:**
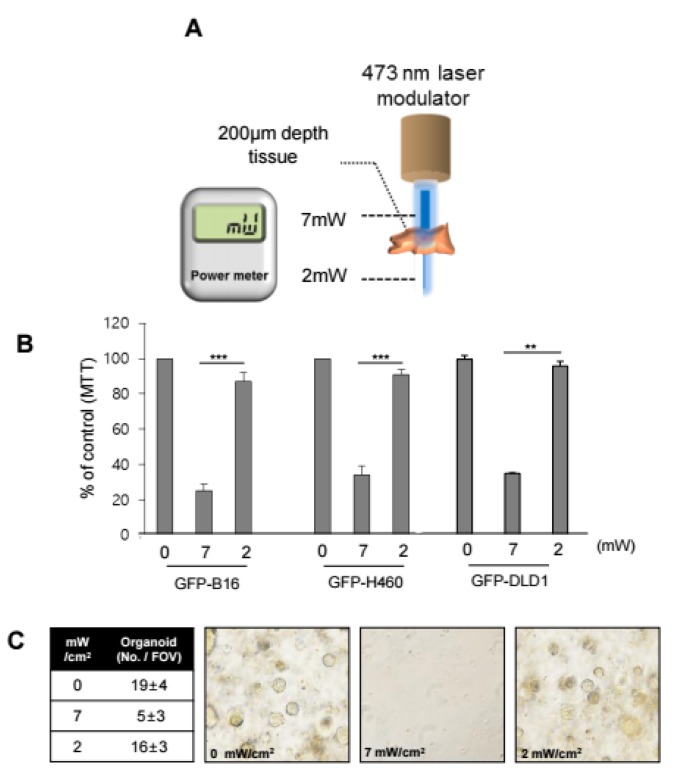
Selection of an appropriate laser power to avoid damage of normal stem cells. (**A**) Photodynamic therapy (PDT) incident light power attenuation was measured with a power meter for transmission through 200 μm of colon tissue. (**B**) Cells survived at 2 mW/cm^2^; however, there was significant cell death at 7 mW/cm^2^ for three types of green fluorescent protein (GFP) cells in vitro. (**C**) Formation and growth of colon organoids were compared after irradiation treatment at 0, 2, and 7 mW/cm^2^. The organoids were counted (upper left panel). The number of organoids did not vary at 2 mW/cm^2^ as compared to the non-treated organoids, but the number significantly decreased following treatment at 7 mW/cm^2^. (40x magnification). ** *p* < 0.01 and *** *p* < 0.001.

For cells sensitized with RB, a series of experiments was performed, which revealed 7 mW/cm^2^ as an appropriate luminance to induce 50% lethal concentration (LC_50_) without significant damage to cells not treated with RB. In our previous report [20], GFP-expressing cells that accumulated RB were more susceptible to 473-nm irradiation than non-GFP cells that accumulated RB. To understand the effect of irradiation through tissue on sensitized GFP+ cells, in vitro colonies were irradiated through several layers of artificial tissue. Artificial tissues of ~50 µm thickness were stacked one by one, and cell death was recorded (Figure S1A, Supplementary Materials). While non-GFP cells survived at rates of ~80% in all cases, GFP+ cell death exceeded 50% until three layers of tissue were used (~150 µm). At ~200 µm of penetration depth, the death rate of GFP+ and non-GFP cells did not vary significantly (Figure S1B, Supplementary Materials). Thus, it was speculated that irradiation at 473 nm would have little effect on cells at the crypt bottom. To further verify the effect, RB was injected intravenously into a mouse xenografted on the right flank with GFP+ lung cancer cell line H460 tumors, which was then exposed to 473-nm light at a power of 7 mW/cm^2^. At a tissue depth of 100–200 µm, histology showed cell death-related hemorrhage (Figure S1C, Supplementary Materials), and cell death markers were strongly stained in the same region (Figure S1D, Supplementary Materials). Seven milliwatts of power from a 473-nm laser was measured before contacting tissue, and ~2 mW penetrating laser power was measured after penetration of the 200-µm tissue in vitro, as shown in Figure 2A.

This attenuation of light intensity was speculated to increase the damage to migrated CSCs located near the luminal surface than to normal colon stem cells located at the crypt bottom. Under illumination at 2 mW and at 7 mW, PDT induced similar cell death patterns in GFP-H460, GFP-B16, and DLD-1 cells (Figure 2B). Furthermore, colonic organoids were prepared from normal Lgr5 mice. Under a 7 mW/cm^2^ treatment regime with RB, limited organoids were formed. In contrast, when the organoids were shielded with 200 μm of artificial tissue, they grew in a manner similar to the naïve control (Figure 2C). This indicated that our PDT targeted only luminal CSCs, and not the normal stem cells in the crypt bottom.

**Figure 3:**
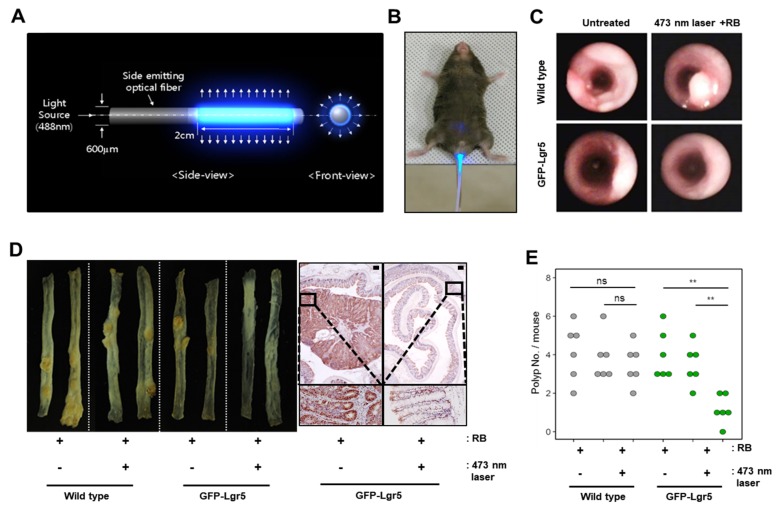
Therapeutic effect of Photodynamic therapy (PDT) on Lgr5+ cells. (**A**) Cylindrical diffuse fibers with a diameter of 600 µm for uniform delivery of light into the mouse colonic epithelium (**B**) Mouse colon irradiation via the anus using cylindrical diffuse fibers. (**C**) Representative in vivo bright-field images of the colon of wild-type (upper column) and green fluorescent protein (GFP)-Lgr5 (lower column) mouse models obtained using the Coloview system. (**D**) Representative images of isolated colons after the experiment. Right panel shows the immunohistochemical analysis of Ki-67 in control (untreated) and laser-irradiated (473-nm laser) colon of GFP-Lgr5 mouse. Scale bar: 200 µm (**E**) Measurement of polyp number under various conditions of PDT. ns, not significant; **, *p* < 0.01. The data presented in the graph indicate a significant reduction in colon polyps when photosensitized cancer stem cells were subjected to PDT treatment.

**Figure 4:**
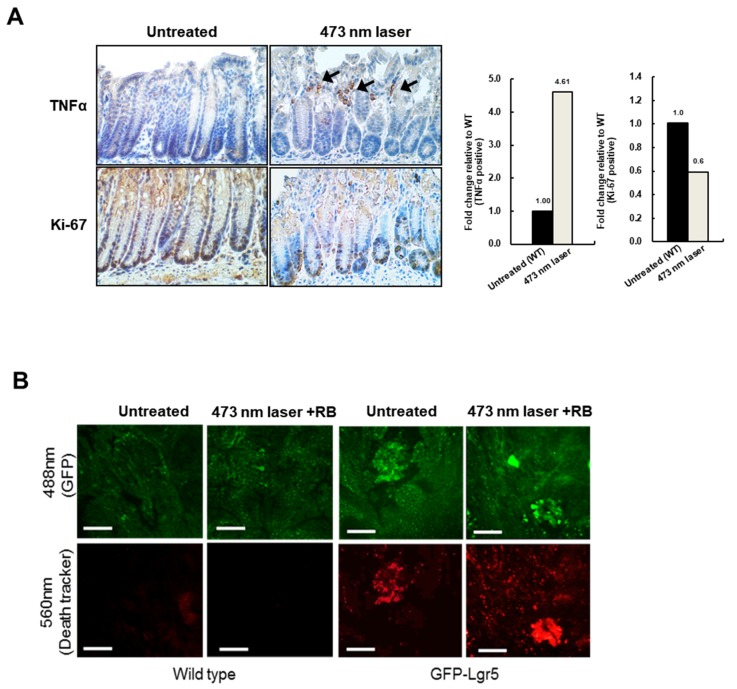
Wavelength-based deep-tissue damage. (**A**) Differences between tumor necrosis factor (TNF)-α and Ki-67 before and after illumination. Arrows indicate TNFα-positive cells. The right panel indicates the q uantitative analysis of Ki-67- and TNFα-positive cells. (**B**) Specific death signal in the circumferential eGFP+ area after rose Bengal (RB) administration and laser irradiation. Scale bar: 100 µm.

To deliver light to the mouse colonic epithelium in vivo, optical fibers that allow uniform radial light emission were manufactured as shown in Figure 3A and were inserted via the anus for PDT (Figure 3B). Colons were treated with blue light in a fractionated course for four hours twice weekly over a seven-week period, following an intravenous injection of RB. After allowing six weeks of tumor formation, whole colons were harvested from both wild-type and GFP-Lgr5 mice. Tumor size was measured by colonoscopy, as shown in Figure 3C. The number of polyps in GFP-Lgr5+ mice was significantly lower than that in wild-type mice (*p* < 0.01) (Figure 3D,E). While, in the luminal area of the crypt, the TNFα signal, which indicates necrotic cell death, was significantly increased in the 473-nm-irradiated mouse, the Ki-67-positive proliferative cells changed only slightly in the bottom region (Figure 4A). Specific cell death observations were also tracked in the circumferential GFP+ area after RB administration and laser irradiation, as shown in Figure 4B.

## 4. Discussion

Targeting CSCs is believed to be an effective therapeutic strategy for the treatment of cancer because CSCs are indispensable in the maintenance and development of cancer. For these reasons, several researchers previously demonstrated the efficacy of CSC-targeted treatment. To eradicate CSC, various approaches including targeting CSC-associated pathways, modulating the CSC microenvironment, gene therapy, and targeting of CSCs themselves are performed [28]. However, the concept of cancer stem cell-specific eradication and its efficacy were not previously demonstrated in a clinical setting, as CSCs are interspersed and share immunological signatures with normal stem cells [1,29,30,31]. For these reasons, selective eradication of cancer stem cells, such that normal stem cells are minimally damaged, is necessary for the clinical application of cancer stem cell-based therapeutic approaches. In addition, efforts are needed to identify unique CSC targets that are not expressed on normal stem cells, or to screen small molecules that can induce differentiation of CSCs [32].

However, by using the novel PDT method and by taking advantage of the localization of cancer stem cells, we selectively eliminated Lgr5+ cells near the colon luminal surface, which are highly likely to be CSCs. In general, photosensitizers, such as RB, accumulate preferentially in tumors due to the enhanced permeability and retention effects [33,34]. However, since RB was injected intravenously before tumors developed into Lgr5-EGFP-IRES-creERT2 knock-in mice that were treated with AOM and DSS, it was distributed equally in Lgr5+ cells that express EGFP and in other cells that do not. When illuminated with a 473-nm blue laser, only EGFP-Lgr5+ cells exhibited fluorescence due to excitation spectrum overlap. The 473-nm wavelength overlaps with the excitation spectrum of EGFP, but not that of RB. As the emission spectrum of EGFP emitted from Lgr5+ cells overlaps with the excitation spectrum of RB, RB inside Lgr5+ cells is activated and produces radical oxygen species and singlet oxygen [35]. Radical oxygen species and singlet oxygen destroy EGFP-Lgr5+ cells selectively; however, non-EGFP cells are minimally affected. To determine the differences in cytotoxicity between EGFP-Lgr5+ cells after laser illumination, we utilized the difference in location between CSCs and normal stem cells. Illumination via the colon lumen resulted in a weakening laser intensity as the beam passed through the colon wall. For this reason, when blue laser was illuminated, EGFPs in the Lgr5+ cells near the colon lumen were fully excited and activated the RB inside Lgr5+ cells. However, EGFPs in the Lgr5+ cells at the crypt bottom were not excited due to the attenuated intensity of the laser. Through these mechanisms and processes, only EGFP-Lgr5+ cells near the colon lumen were selectively destroyed with minimal damage to EGFP-Lgr5+ cells at crypt bottom and non-EGFP cells.

Hereditary colorectal cancer syndromes, such as familial adenomatous polyposis (FAP) and Lynch syndrome, account for about 5–6% of all colorectal cancers [36]. Due to the high cancer incidence rate, colonoscopy should be performed every 1–2 years starting at the age of 10–11 years in families with classic familial adenomatous polyposis (FAP) [37,38]. In Lynch syndrome patients, colonoscopy should be conducted every 1–2 years starting at the age of 20–25 years [39,40]. Despite repeated surveillance, more than half of the hereditary colorectal cancer syndrome patients suffer from colorectal cancer and require surgery [36]. In particular, the lifetime risk of colorectal cancer in classic FAP patients is over 90% [37]. When FAP patients have a large number of adenomas, including high-grade dysplasia, proctocolectomy with ileac pouch anal anastomosis is needed [40]. Considering that most FAP patients undergo surgery at a young age, the patients are highly inconvenienced by the need to live with a colostomy bag for the rest of their lives. With modifications made for the human colon, as discussed below, the proposed treatment, combining photosensitizers, PDT, and Lgr5+ cells, promises to help improve the quality of life for hereditary colorectal cancer syndrome patients. By enabling treatment to prevent the formation of early-stage colon polyps, the incidence of colon cancer and the required number of surgeries may be reduced. 

The location of normal Lgr5+ cells at crypt base in humans is deeper than in mice, at over 500 μm from the luminal surface [10]. Due to the shallow optical penetration depth in the lower visible spectrum, PDT using the combination of RB and 473-nm illumination is not appropriate. Furthermore, since human Lgr5+ cells do not express GFP, the present PDT method cannot be directly utilized in a clinical setting. To adopt localized targeting of Lgr5+ cancer stem cells for human patients, a redesign of the PDT system is necessary. 

One possibility for clinical adaptation would combine a high-penetration-depth near-infrared (NIR) illumination source, second-generation NIR-activated photosensitizers such as Chlorin e6 (Ce6), and conjugated Lgr5 antibodies. Although the penetration depth of NIR lasers can be 1–2 mm, the NIR wavelength region is the most suitable after considering energy losses. In addition, although present second-generation photosensitizers conjugated to Lgr5 antibodies bind to both cancer stem cells and normal stem cells, NIR illumination using depth-based treatment localization as demonstrated here would enable the selective targeting of cancer stem cells up to 500 μm from the luminal surface. The shift to near-infrared wavelengths would also minimize trauma to non-photosensitized cells and tissues. Depth-based selectivity may also be combined with other methods, such as using photosensitizers and upconversion nanoparticles conjugated with antibodies against more selective cancer stem markers like Dclk1, to overcome the limitations of laser penetration depth and to further enhance selective toxicity against cancer stem cells [41,42].

## 5. Conclusions

In conclusion, we presented a novel PDT method which demonstrates the potential for selective, localized targeting of CSCs based on the penetration depth of light used for PDT, using the example of Lgr5+ CSCs in a GFP-Lgr5+ transgenic mouse model to demonstrate the concept. The novel PDT method inhibited tumor growth and reduced the number of polyps significantly in a model of inflammation-induced colorectal cancer, while minimizing damage to normal stem cells which were also photosensitized. Based on this finding, we provide additional circumstantial evidence that Lgr5+ cells near the colonic luminal surface are likely to be CSCs. This strategy of localized targeting may be pursued to prevent the development of precancerous lesions and colon cancer in hereditary colorectal cancer syndromes, with the goal of avoiding extensive surgeries. In combination with the development of novel cancer stem-cell markers, this novel PDT technique might serve as a cancer prevention and treatment method for high-risk patients.

## Supplementary Materials

The following are available online at https://www.mdpi.com/2072-6694/12/1/203/s1: Figure S1: Tissue depth dependent attenuation of light energy; Video S1: Z-stack images of Lgr5+ GFP cells inside adenoma induced by AOM and DSS.
