# Supplementary material for: Selective Targeting of Cancer Stem Cells (CSCs) Based on Photodynamic Therapy (PDT) Penetration Depth Inhibits Colon Polyp Formation in Mice
Source: Cancers (Basel). 2020 Jan 14;12(1):203. doi: 10.3390/cancers12010203 (PMC7017170; doi:10.3390/cancers12010203)
Supplement: Supplementary file 1 [file cancers-12-00203-s001.zip › cancers-659532-supplementary/cancers-659532-supplementary final.docx]

**Supplementary Materials**


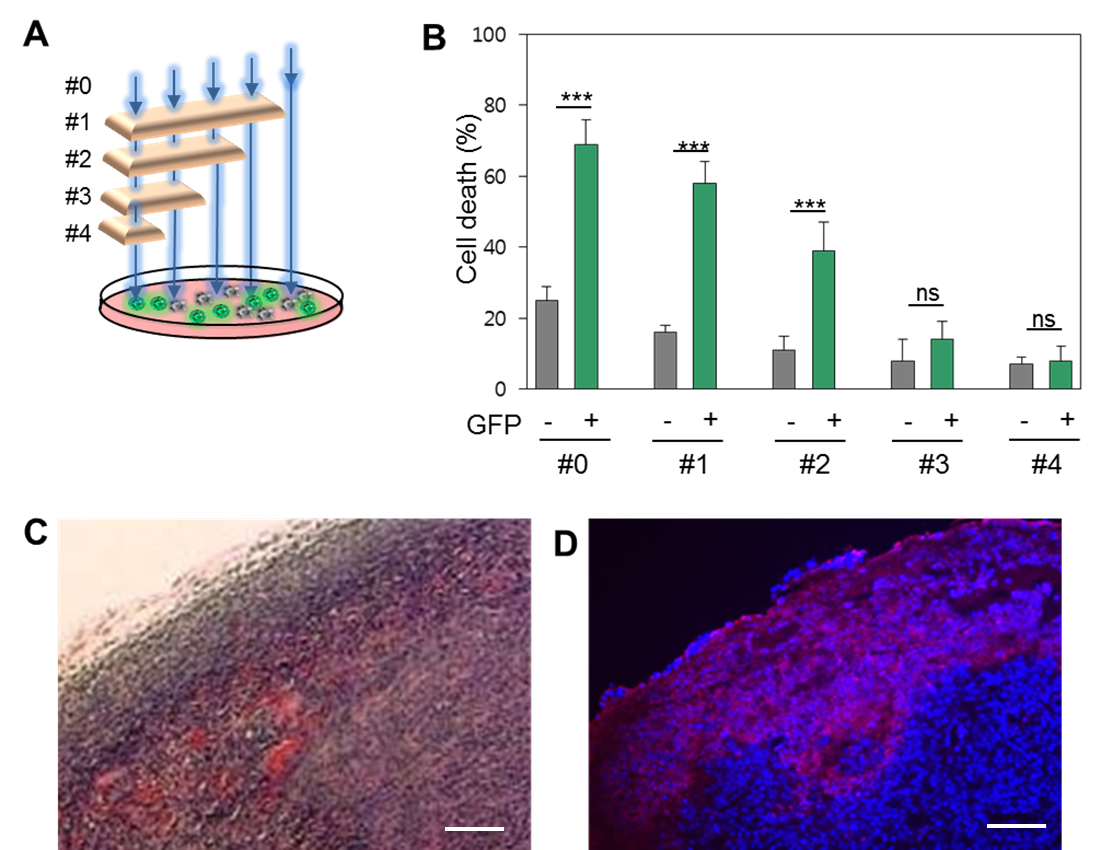


**Figure S1.** Tissue depth dependent attenuation of light energy. (**A**) Schematic explanation of layering 50-μm-thick artificial tissues to attenuate light power and to mimic in vivo conditions. (**B**) Cell death of GFP and non-GFP cells was compared in vitro after irradiation through tissue layers of each depth; ***, *p* < 0.001. (**C**) H&E staining in tumor xenografted from H460 cells after 473-nm irradiation. (**D**) Cell death monitored with immunofluorescent staining on TNFα. Scale bar: 100 µm

Methods for Supplementary figure S1:

Depth dependent cell death

Control H460 and GFP expressed H460 cells were seeded on 6-well culture plate and incubated with 100 nM RB for 4 h. And then, the cells were irradiated a light that the intensity was reduced by stacking of artificial tissues. The artificial tissue containing collagen matrix were used to simulate the attenuation of light though different tissue depths. Then, the cell viability was measured using MTT assay. GFP-H460 cells were subcutaneously injected into nude mice, formed tumor, treated RB and isolated tumor tissue. And then, isolated tissue was cut at 10 μm thickness and stained with antibodies or H&E.

Supplementary Movie legends

**Movie S1.** Z-stack images of Lgr5^+^ GFP cells inside adenoma induced by AOM and DSS. The fluorescent cells are Lgr5^+^ GFP cells, the upper left area of the frame represents the colonic lumen, while the lower right area represents the bottom of the crypt.
