# Supplementary material for: Selective Targeting of Cancer Stem Cells (CSCs) Based on Photodynamic Therapy (PDT) Penetration Depth Inhibits Colon Polyp Formation in Mice
Source: Cancers (Basel). 2020 Jan 14;12(1):203. doi: 10.3390/cancers12010203 (PMC7017170; doi:10.3390/cancers12010203)
Supplement: Supplementary file 1 [file cancers-12-00203-s001.zip › ijerph-661783.docx]

Article

Built and Natural Environmental Correlates of Parental Safety Concerns for Children’s Active Travel to School

Young-Jae Kim ^1,^* and Chanam Lee ^2^

^1^ Department of Forest Resources and Landscape Architecture, Yeungnam University, 280 Daehak-Ro, Gyeongsan, Gyeongbuk 38541, Korea

^2^ Department of Landscape Architecture and Urban Planning, Texas A&M University, 3137 TAMU, College Station, TX 77843-3137, USA; chanam@tamu.edu

***** Correspondence: youngjae_kim@yu.ac.kr; Tel.: +82-53-810-2978

Received: 21 November 2019; Accepted: 2 January 2020; Published: date

**Abstract:** This cross-sectional study examines built and natural environmental correlates of parental safety concerns for children’s active travel to school (ATS), controlling for socio-demographic, attitudinal, and social factors. Questionnaire surveys (*n* = 3291) completed by parents who had 1st–6th grade children were collected in 2011 from 20 elementary schools in Austin, Texas. Objectively-measured built and natural environmental data were derived from two software programs: Geographic Information Systems (GIS) and Environment for Visualizing Images (ENVI). Ordinal least square regressions were used for statistical analyses in this study. Results from the fully adjusted final model showed that bike lanes, the presence of highway and railroads, the presence of sex offenders, and steep slopes along the home-to-school route were associated with increased parental safety concerns, while greater intersection density and greater tree canopy coverage along the route were associated with decreased parental safety concerns. Natural elements and walking-friendly elements of the built environment appear important in reducing parental safety concerns, which is a necessary step toward promoting children’s ATS.

**Keywords:** active travel to school; home-to-school route; built and natural environment; parental safety concerns; children

1. Introduction

Physical activity is important for children’s health, and the U.S. Department of Health and Human Services issued the Physical Activity Guidelines for Americans in 2008 that recommends 60 minutes or more of daily physical activity for children [1]. Most children in the U.S., however, do not meet this recommendation. As a feasible way to increase children’s daily physical activity levels, active travel to school (ATS) by walking and bicycling has received wide attention from the public. Evidence from many empirical studies confirms that ATS among school-aged children contributes to increasing their overall physical activity levels [2–4]. Despite its health-significant roles, ATS has continued to decrease over the last few decades, from 47.7% in 1969 to 12.7% in 2009 [5].

To identify the reasons why some children walk or bicycle to school but others do not, a number of studies examined and confirmed the roles of environmental factors such as roadway conditions, sidewalk availability, transportation infrastructure, land uses, and urban form [6–9]. Most previous studies have examined how individual environmental variables are positively or negatively associated with ATS behaviors. However, children’s ATS is determined primarily by their parents, and parental decisions about their child’s school travel mode are based on a wide range of personal and environmental factors, especially those related to safety. Due to the difficulty in changing the personal factors, addressing the modifiable environmental factors that can help reduce parental safety concerns is important for developing effective intervention strategies to promote children’s ATS [10].

In the HealthStyles survey by the Centers for Disease Control and Prevention in 2002, safety issues related to traffic- and crime-danger were ranked as the second most frequently reported barrier to ATS among parents, followed only by the long distance barrier. While the distance barrier is critical to ATS, it requires a long term and multi-level policy and environmental changes. Safety related barriers are related to environmental conditions (e.g., sidewalks, crosswalks, and traffic) that are more readily modifiable through proper planning or design strategies. Therefore, understanding those modifiable environmental features associated with parental safety concerns can lead to more immediately implementable intervention strategies that can reduce parental barriers to children’s ATS.

Previous studies have already documented that parental safety concerns are the main impediment to children’s ATS. The specific variables related to parental safety concerns that have been studied to influence children’s ATS include neighborhood safety problems [11,12], strangers in neighborhoods [12], traffic dangers [12–14], and walking-hostile transportation infrastructure such as highways or railroads [14]. A recent study conducted by Bennets et al. showed that parental fear about children’s independent mobility was associated with perceived disapproval from others and parents’ perception of children’s competence to travel safely [15]. Although a number of studies have identified the relationships between various parental safety perception variables and children’s ATS, little is known about how parental safety concerns are associated with surrounding environmental conditions. A recent study showed that parents’ perceptions about safety from traffic in a neighborhood environment were not associated with objectively assessed characteristics [16]. In contrast, Lee and Kim [17] found that parental safety concern was an intermediate outcome between the built environment and children’s walking to school, and that parents’ perceived measures and objective environmental measures are significantly correlated. Using structural equation modeling, their study showed that destination land uses, non-residential land uses, and walking-hostile infrastructure such as highways and busy traffic roads, increased parental safety concerns and were indirectly associated with children’s walking to school through their influences on parental safety concerns. The direct link between parental safety concern and children’s walking to school was not found in their study, further suggesting the significant and potentially determinant role of parental safety concerns in children’s school travel mode choice. More studies are needed to better understand the specific environmental features that may contribute to reducing or increasing parental safety concerns and to identify relevant intervention strategies that can effectively target this important parental barrier as a prerequisite to ATS promotion.

Given the shortage of previous studies that specifically focus on parental perceptions of safety related to children’s ATS, this study examines if and how built and natural environmental conditions along the home-to-school travel route are associated with parental safety concerns, controlling for personal, attitudinal, and social factors. Compared to the previous studies focusing primarily on the built environmental variables, the consideration of both built and natural environmental factors is another important contribution of this study to the existing literature on this topic. Further, the objective measurement of the environmental variables along the likely travel route between home and school increases the specificity of the environmental exposure to better match with the outcome of parental safety concerns, which were specifically asked for school commuting in this study.

2. Materials and Methods

2.1. Study Setting and Data Collection

The study area is the Austin Independent School District (AISD), which covers most of the City of Austin, Texas. The 20 elementary schools from the total of 81 schools in the AISD were selected for this study based on the following four criteria: (1) school location (i.e., spatial distribution), (2) environmental settings (i.e., inner-city, urban, and suburban, based on its proximity to downtown and neighborhood characteristics), (3) socio-economic status, and (4) research approval from AISD and the individual schools. The selected study schools and attendance areas represent a wide range of economic and environmental conditions to help assess their roles in influencing parental safety concerns related to ATS

Out of 13,573 initial paper-surveys sent to the 20 selected schools, 4609 parents’ surveys were returned (response rate = 34.0%). Among the returned surveys, 4270 reported a home address that could be geocoded, which was necessary to generate the home-to-school route measure. Finally, after further excluding those who did not provide valid/complete survey data needed for this study, 3291 parental surveys were included in the analysis for this study. The study protocol was approved by AISD and the Texas A&M University Institutional Review Board (#2010-0358). More information about the study methods, including the detailed recruitment and data collection process, can be found elsewhere [18].

The survey included questions about socio-demographic, attitudinal and social factors, and environmental perceptions that are associated with ATS. The environmental variables are also measured objectively. The Geographic Information System (GIS) software used to capture the built environmental measures, and the Environment for Visualizing Images (ENVI) software was used to generate the natural environmental variables.

2.2. Dependent Variable: Parental Concerns about Safety

Parental safety concerns about ATS were measured based on the responses to the eight survey questions: (a) my child may get lost, (b) my child may be taken or hurt by a stranger, (c) my child may get bullied, teased, or harassed, (d) my child may be attacked by stray dogs, (e) my child may be hit by a car, (f) exhaust fumes may harm my child’s health, (g) no one will be able to see and help my child in case of danger, and (h) my child may get injured by falling (e.g., due to drainage ditches, uneven walking surfaces). These items were adopted and developed from existing survey measures and previous studies [19–21]. The response option to these survey items was a 5-point Likert scale coded from 1 for “strongly disagree” to 5 for ”strongly agree”. Higher scores indicate greater parental safety concerns for children’s ATS. To generate the safety concern outcome variable, a composite measure was used to compute the mean of the eight Likert-scale items, and the output was treated as a continuous variable in the statistical analysis [22]. Principal-component factor analysis based on the eight survey items loaded showed only one factor having an Eigenvalue of greater than 1 (Eigenvalue = 4.54) and showed that all the factor loadings were greater than 0.7, supporting the validity of combining the eight items into one composite scale variable [23].

2.3. Independent Variables: Built and Natural Environments

The objectively-measured built environmental variables included transportation infrastructure conditions (e.g., bike lanes, sidewalks, highways, railroads, intersections) collected from the Texas Department of Transportation, crime types and locations from the Texas Department of Public Safety, crash locations from the Austin Police Department, and playgrounds and traffic volume from the City of Austin. For the crime and crash variables, a hotspot analysis (expressed with z-scores) was undertaken using a model builder in ArcGIS. The statistically significant positive z-scores of the crime and crash variables indicate more intense clustering of high values (hot spot) [24].

The objectively-measured natural environmental variables included parks (presence and amount within the home-to-school route buffer) from the City of Austin, steep slopes (on average derived from a digital elevation model), land cover types (urbanized area, tree canopy, and grass cover) classified from a digital orthophoto quarter quadrangle image through ENVI software, tree heights derived from light detection and ranging, and surface temperature and the normalized difference vegetation index (NDVI) derived from a remotely-sensed image from Landsat 5TM.

After estimating the shortest home-to-school route through a network analysis based on the geocoded home and school locations in ArcGIS, all the built and natural environmental variables were measured within the 200-feet home-to-school route buffer (Figure 1). Because the survey questions used to measure the parental safety concern outcome variable were specifically about their children’s walking from home to school, the use of a route-based buffer helps match the spatial extent/exposure of the objective measures with that of the survey. The buffer width of 200 feet was selected as an appropriate distance to capture the environment proximal to the road [9]. Further, our internal tests with similar datasets showed that this is an optimal width to reduce overlaps among different home-to-school routes while still capturing all the relevant roadside and land-use measures for this type of study.


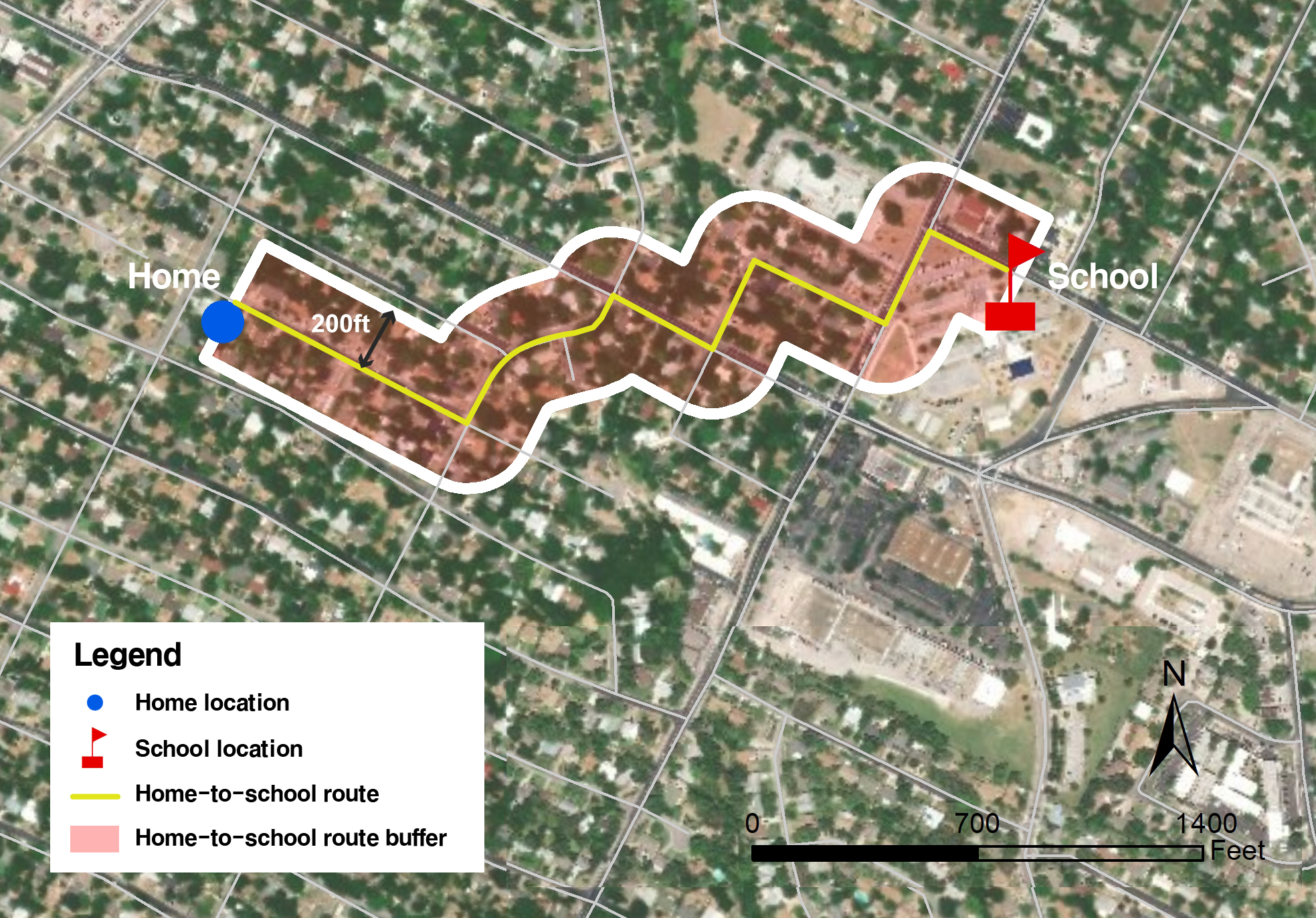


**Figure 1.** Built and natural environment measurements with home-to-school route buffer of 200 feet.

2.4. Confounding Factors: Socio-Demographic, Attitudinal, and Social Factors

A number of socio-demographic, attitudinal, and social factors shown to be significant in previous studies were considered as potential confounding variables in this study [7,25–29], which were later used to build the base model during the modeling process. Socio-demographic factors included student gender, grade, and ethnicity. The household income variable had a significant number (*n* = 1315, 40% out of 3291) of missing values, and therefore a dummy variable indicating whether or not students received free or reduced-price school lunch was used as a proxy of income status. The survey questions about attitudinal and social factors had a 5-point Likert scale with the response options from “strongly disagree” to “strongly agree”. The four attitudinal factors considered in this study were (a) Walking is a good way to exercise, (b) I walk quite often in my daily routine, (c) I (would) enjoy walking with my child to/from school, and (d) My family and friends like the idea of walking to school. Social factors were measured with two items: (a) Other kids walk to/from school in my neighborhood, and (b) I feel connected to people in my neighborhood.

2.5. Data Analysis and Modeling Process

Statistical analyses were undertaken using ver. 12 STATA (StataCorp LLC, College Station, TX, USA) [30] to examine the relationships between the environmental variables and parental concerns about the safety of their children while traveling to school. An ordinary least square (OLS) regression model was used for the continuous outcome variable (mean of eight Likert-scale survey items).

The regression modeling involved the following five steps (Figure 2). First, a base model including all significant personal, attitudinal, and social factors was generated for the outcome variable. Second, each built and natural environmental variable was added to the base model one at a time (one-by-one tests). Third, all significant built environmental (BE) variables from the one-by-one tests were added together to the base model to develop the BE model, which included the most significant built environmental variables after considering their theoretical significance. Fourth, utilizing all significant natural environmental (NE) variables from the one-by-one tests, the same process was applied to generate the NE model. Finally, all significant variables from the BE and NE models were added together to generate the final model, which included all significant built and natural environmental variables at the 0.05 level.

**Figure 2.** Built and natural environment measurements with home-to-school route buffer of 200 feet.

3. Results

3.1. Sample Characteristics

Table 1 shows the sample characteristics. Students were gender-balanced (51.4% female, 48.6% male), and their grade levels included pre-kindergarten—kindergarten (PK–K, 26.6%), 1st–3rd (49.3%) and 4th–6th (24.1%). Those from the 7th grade or higher were excluded due to the small sample cases (*n* = 15, 0.3%). The child’s ethnicity was categorized as non-Hispanic white (27.0%), Hispanic (62.3%), and others (10.8%). About 63.2% of students qualified for the special (reduced price or free) school lunch program and were considered as a low-income group in this study.

**Table 1.** Descriptive statistics of outcome variable and confounding variables.

| **Variables** | ***n*** | **Mean** | **SD** | |
| --- | --- | --- | --- | --- |
| Parental safety concerns ^†^ (outcome variable)  (mean score of following 8 items with a 5-point Likert scale) | 3291 | 3.28 | 1.03 | |
| (a) My child may get lost. | 3243 | 2.96 | 1.52 | |
| (b) My child may be taken or hurt by a stranger. | 3258 | 3.77 | 1.31 | |
| (c) My child may get bullied, teased, or harassed. | 3241 | 3.27 | 1.38 | |
| (d) My child may be attacked by stray dogs. | 3253 | 3.30 | 1.41 | |
| (e) My child may be hit by a car. | 3251 | 3.90 | 1.29 | |
| (f) Exhaust fumes may harm my child’s health. | 3222 | 2.93 | 1.34 | |
| (g) No one will be able to see and help my child in case of danger. | 3235 | 3.19 | 1.35 | |
| (h) My child may get injured by falling (due to drainage ditches, uneven walking surfaces, etc.). | 3242 | 2.95 | 1.39 | |
| Socio-demographic factors ^‡^ (confounding variables) |  |  |  | |
| Student Gender | 0: Female (1697, 51.4%), 1: Male (1607, 48.6%) | | | |
| Grade | 1: PK—K (878, 26.6%), 2: 1st—3rd (1629, 49.3%), 3: 4th—6th (797, 24.1%) | | | |
| Free or Reduced Lunch Qualification | 0: No (1216, 36.8%), 1: Yes (2088, 63.2%) | | | |
| Student Ethnicity | 1: Non-Hispanic White (891, 27.0%), 2: Hispanic (2057, 62.3%), 3: Others (356, 10.8%) | | | |
| Attitudinal and social factors ^†‡^ (confounding variables) |  |  | |  |
| Walking is a good way to exercise. | 3291 | 4.78 | | 0.66 |
| I walk quite often in my daily routine. | 3291 | 3.93 | | 1.19 |
| I (would) enjoy walking with my child to/from school. | 3291 | 4.11 | | 1.20 |
| My family and friends like the idea of walking to school. | 3291 | 3.74 | | 1.27 |
| Other kids walk to/from school in my neighborhood. | 3291 | 3.63 | | 1.48 |
| I feel connected to people in my neighborhood. | 3291 | 3.72 | | 1.25 |

Note: ^†^ These variables indicate the respondents’ feelings about walking and their neighborhood, and were treated as continuous variables coded by 1 for “strongly disagree”, 2 for “somewhat disagree” , 3 for “neither disagree nor agree”, 4 for “somewhat agree”, and 5 for “strongly agree”. ^‡^ These variables were used as confounding factors for the final regression models. SD—standard deviation.

3.2. Correlates of Parental Safety Concerns

Table 2 shows the results from the OLS regressions estimating the built and natural environmental correlates of parental safety concerns, controlling for the socio-demographic, attitudinal, and social confounders. The table includes the results from the one-by-one tests, the BE model, the NE model, and the Final Model.

**Table 2.** Built and natural environmental correlates of parental safety concerns (*n* = 3291).

| **Safety Concern**  **(OLS Regression)** | **One-by-One Tests** | | **BE Model** | | **NE Model** | | **Final Model** | | | |
| --- | --- | --- | --- | --- | --- | --- | --- | --- | --- | --- |
|  | **Coef.** | ***p*** | **Coef.** | ***p*** | **Coef.** | ***p*** | **Coef.** | ***p*** | **95% Cl** | |
|  |  |  |  |  |  |  |  |  | **Low** | **High** |
| Built environmental variables |  |  |  |  |  |  |  |  |  |  |
| Bike lanes (ratio) | 0.123 | 0.035 | 0.131 | 0.027 |  |  | 0.107 | 0.073 | −0.010 | 0.223 |
| Sidewalks (ratio) | −0.222 | 0.011 | −0.310 | 0.002 |  |  | - | - | - | - |
| Playgrounds (presence) | 0.018 | 0.748 | - | - |  |  | - | - | - | - |
| Intersections (density, num./acre) | −0.728 | 0.001 | −0.741 | 0.001 |  |  | −0.642 | 0.003 | −1.061 | −0.222 |
| Highways (presence) | 0.204 | 0.000 | 0.163 | 0.001 |  |  | 0.099 | 0.051 | −0.001 | 0.199 |
| Railroads (presence) | 0.240 | 0.000 | 0.127 | 0.024 |  |  | 0.136 | 0.019 | 0.022 | 0.250 |
| High speed streets (>30 mph) (%) | 0.002 | 0.014 | - | - |  |  | - | - | - | - |
| Crime hotspot (z-scores) | 0.136 | 0.000 | 0.117 | 0.000 |  |  | - | - | - | - |
| Crash hotspot (z-scores) | 0.016 | 0.002 | - | - |  |  | - | - | - | - |
| Sex offenders (presence) | 0.236 | 0.000 | 0.143 | 0.004 |  |  | 0.145 | 0.001 | 0.056 | 0.234 |
| Natural environmental variables | | | | | | | | | | |
| Park (presence) | 0.082 | 0.031 |  |  | - | - | - | - | - | - |
| Water feature (presence) | 0.251 | 0.000 |  |  | 0.214 | 0.000 | 0.117 | 0.007 | 0.031 | 0.202 |
| Mean slope (degrees) | 0.027 | 0.000 |  |  | 0.031 | 0.000 | 0.034 | 0.000 | 0.020 | 0.049 |
| Urbanized area (%) | 0.004 | 0.048 |  |  | - | - | - | - |  |  |
| Tree canopy (%) | −0.016 | 0.000 |  |  | −0.017 | 0.000 | −0.014 | 0.000 | −0.021 | −0.007 |
| Grass cover (%) | −0.001 | 0.785 |  |  | - | - | - | - | - | - |
| Surface temperature (%) | −0.018 | 0.121 |  |  | - | - | - | - | - | - |
| Normalized Difference Vegetation Index (NDVI) | −0.010 | 0.000 |  |  | - | - | - | - | - | - |
| Tree heights (feet) | −0.017 | 0.000 |  |  | - | - | - | - | - | - |
| Socio-demographic variables ^†^ |  |  |  |  |  |  |  |  |  |  |
| Student gender (male vs. ref. female) | |  | −0.046 | 0.166 | −0.052 | 0.123 | −0.048 | 0.149 | −0.114 | 0.017 |
| Student grade (ref. PK–K) |  |  |  |  |  |  |  |  |  |  |
| 1st–3rd |  |  | −0.092 | 0.024 | −0.089 | 0.029 | −0.095 | 0.019 | −0.174 | −0.015 |
| 4th–6th |  |  | −0.214 | 0.000 | −0.220 | 0.000 | −0.223 | 0.000 | −0.315 | −0.130 |
| Free or reduced lunch (yes vs. ref. no) | |  | 0.095 | 0.083 | 0.096 | 0.059 | 0.105 | 0.043 | 0.003 | 0.206 |
| Student ethnicity (ref. White) |  |  |  |  |  |  |  |  |  |  |
| Hispanic |  |  | 0.235 | 0.000 | 0.229 | 0.000 | 0.242 | 0.000 | 0.135 | 0.348 |
| Others |  |  | 0.032 | 0.618 | 0.033 | 0.611 | 0.033 | 0.617 | −0.095 | 0.160 |
| Attitudinal/social variables ^†^ |  |  |  |  |  |  |  |  |  |  |
| Walking is a good way to exercise. | |  | 0.249 | 0.000 | 0.255 | 0.000 | 0.251 | 0.000 | 0.197 | 0.304 |
| I walk quite often in my daily routine. | |  | 0.052 | 0.001 | 0.051 | 0.001 | 0.050 | 0.001 | 0.019 | 0.081 |
| I (would) enjoy walking with my child to/from school. |  |  | −0.055 | 0.004 | −0.054 | 0.004 | −0.053 | 0.005 | −0.090 | −0.016 |
| My family and friends like the idea of walking to school. |  |  | −0.045 | 0.015 | −0.046 | 0.014 | −0.045 | 0.016 | −0.081 | −0.008 |
| Other kids walk to/from school in my neighborhood. |  |  | −0.056 | 0.000 | −0.063 | 0.000 | −0.054 | 0.000 | −0.080 | −0.027 |
| I feel connected to people in my neighborhood. |  |  | −0.092 | 0.000 | −0.091 | 0.000 | −0.090 | 0.000 | −0.120 | −0.061 |

Note: The one-by-one model indicates a model estimated by which an environmental variable was entered one at a time into the model, including all the covariates. Values of all the covariates, total *n* and R2 generated from each one-by-one model were not included in the table due to space considerations. ^†^ The covariates were socio-demographic, attitudinal, and social variables. OLS—ordinary least square; NE—natural environment; BE—built environment; Coef.—coefficient; CI—confidence interval.

3.2.1. Personal, Attitudinal, and Social Correlates of Parental Safety Concerns

All the covariates across the model presented similar results in terms of the statistical significance and the direction of association with the outcome. Thus, we focus our discussions here on the results from the final model, which includes both the built and natural environmental variables.

Parents with older children (Grades 1–3 or 4–6) had lower safety concerns than those with younger ones at the pre-kindergarten or kindergarten level (Coef. = −0.095, *p* = 0.019 for 1st–3rd grade, Coef. = −0.223, *p* < 0.001 for 4th–6th grade). Parents whose children were Hispanic and received free or reduced-price school lunch had higher safety concerns than parents whose children were non-Hispanic white and were not eligible for the special lunch service (Coef. = 0.242, *p* < 0.001 for Hispanic, Coef. = 0.105, *p* = 0.043 for special lunch).

The two attitudinal variables, “walking is a good way to exercise (Coef. = 0.251, *p* < 0.001)” and “I walk quite often in my daily routine (Coef. = 0.050, *p* = 0.001)”, were associated with increased parental safety concerns. In contrast, positive responses to the four social factors, “I (would) enjoy walking with my child to/from school (Coef. = −0.053, *p* = 0.005)”, “My family and friends like the idea of walking to school (Coef. = −0.045, *p* = 0.016)”, “Other kids walk to/from school in my neighborhood (Coef. = −0.054, *p* < 0.001)”, and “I feel connected to people in my neighborhood (Coef. = −0.090, *p* < 0.001)”, were correlated with reduced safety concerns.

3.2.2. Built and Natural Environmental Correlates of Parental Safety Concerns

The BE model shown in Table 2 presents the results from the regression model that identified significant “built” environmental variables associated with parental safety concerns, controlling for the socio-demographic, attitudinal, and social variables (R^2^ = 0.1349). Significant built environmental correlates of parental safety concern included bike lane ratios (Coef. = 0.131, *p* = 0.027), sidewalk ratios (Coef. = −0.310, *p* = 0.002), intersection density (Coef. = −0.741, *p* = 0.001), the presence of highways (Coef. = 0.163, *p* = 0.001), the presence of railroads (Coef. = 0.127, *p* = 0.024), crime hotspots (Coef. = 0.117, *p* < 0.001), and presence of sex-offenders (Coef. = 0.143, *p* = 0.004). The NE model in Table 2 showed significant “natural” environmental variables associated with parental safety concerns, controlling for the covariates (R^2^ = 0.1315), which included water features (Coef. = 0.214, *p* < 0.001), mean slopes (Coef. = 0.031, *p* < 0.001), and tree canopy (Coef. = −0.017, *p* < 0.001).

The final model was generated by combining the BE model with the NE model and retaining only the significant variables. The predictor variables included in the final model accounted for 14.1% of the variance in the parental safety concern variable, which was slightly higher than the variances accounted for in the BE model (13.5%) and the NE model (13.2%). The statistical significance of sidewalk ratios, presence of highways and crime hotspot variables in the BE model disappeared in the final model, while the three natural environmental variables associated with parental safety concerns in the NE model retained their statistical significance in the final model. Based on the results of the final model, more bike lanes (Coef. = 0.107, *p* = 0.073), the presence of highways (Coef. = 0.099, *p* = 0.051), the presence of railroads (Coef. = 0.136, *p* = 0.019), and the presence of sex-offender home locations (Coef. = 0.145, *p* = 0.001) along the home-to-school route were associated with increased parental safety concerns, while higher street intersection density representing better connectivity (Coef. = −0.642, *p* = 0.003) was correlated with decreased safety concerns. Among the natural environmental variables, the presence of water features (Coef. = 0.117, *p* = 0.007) and greater steep slopes (Coef. = 0.034, *p* < 0.001) were positively associated with parental safety concerns while greater tree canopy indicating better shade conditions (Coef. = −0.014, *p* < 0.001) was negatively associated with parental safety concerns.

4. Discussion

ATS is considered a promising way to help children incorporate physical activity into their daily routines. However, many barriers have kept them from ATS and one of the leading barriers is related to the safety problems perceived by the parents. As the primary decision-maker of a child’s school travel mode choice, addressing such parental barriers is the key to promote children’s ATS. To fill in the knowledge gap in the ATS literature, this research focused on environmental factors related to parental safety concerns related to ATS.

With the use of objective measures in GIS and ENVI, both built and natural environmental features were examined and a number of modifiable features significantly correlated with parental safety concerns were identified. Many features along the home-to-school route were shown to be associated with increased safety concerns, which included four BE variables (bike lanes, highways, railroads, and sex-offender home locations), and two NE variables (water features, and steep slope areas). However, only one BE variable (street connectivity) and one NE variable (tree canopy area) were associated with decreased parental safety concerns. Among the significant environmental correlates, the bike lane ratio variable (associated with increased parental safety concern) may appear counterintuitive. However, while bike lanes are important for promoting bicycling and possibly other types of active transportation activity for adults [31], they may act as a barrier to ATS among children because parents may have a fear of bicyclists due to the high vulnerability of child pedestrians. Most bike lanes in Austin and many other cities in the US are located along vehicular roadways with simple painted/striped lines without any physical buffers from cars or pedestrians, and thus increasing parental safety concerns especially for the young children. Further, this study supports the findings from several previous studies related to children’s ATS. Ewing et al. [32,33] and Dalton et al. [6] showed that higher sidewalk coverage increased the odds of ATS. This study found that an increase in sidewalk coverage led to a significant decrease in parental safety concerns. Related to the findings of several studies identifying that children were more likely to walk or bike to school in a neighborhood where street connectivity was high [34,35], this study also showed that higher street connectivity reduced parental safety concerns. Several studies including those conducted by Scholossberg et al. [36] and Zhu and Lee [14] showed that the presence of railroad tracks and highways were negative correlates of children walking to school. This study further showed that those barriers to walking to school were also related to increased parental safety concerns. Regarding the natural environmental variables, this study showed that greater tree canopy coverage present along the home-to-school route was associated with decreased parental safety concerns, while steep slope areas were related to increased safety concerns. This finding is consistent with the findings from ATS studies showing positive relationships between trees in the neighborhood and ATS [7], and between gentle/flat terrain and ATS [29].

Regarding the socio-demographic, attitudinal, and social factors used as the covariates in this study, parents whose children were from higher grades had less safety concerns for their children’s ATS while parents whose children were Hispanic had greater safety concerns than parents whose children were white. Further, parents whose children received free or reduced-price lunch at school had higher safety concerns than their counterparts. These findings suggest that environmental conditions in areas where minority and low-income households live are less desirable and less walking-friendly (e.g., more crime and crash incidents, fewer sidewalks, fewer trees) leading to greater parental safety concerns for their children’s ATS in this study. This study also showed that the two attitudinal factors, including “walking is a good way to exercise” and “I walk quite often in my daily routine” increased parental safety concerns. This finding can be explained by the likelihood that parents who walk more often in their daily routine and those who have positive attitudes toward walking may have more concerns about safety because they actually walk and therefore notice more problems/conditions in the environment that can be potentially harmful to their children. This point is associated with a causality issue, but as a cross-sectional study, we were unable to explore causal relationships among the study variables. Future studies need to consider the causal relationships between the study variables and to explore the detailed mechanisms underlying these relationships. In contrast to the associations between the attitudinal factors and parental safety concerns, this study showed that parental safety concerns decreased if parents had more positive perceptions of social environments (e.g., their family/friends/other kids’ walking to school) and felt a stronger social connection with people in their neighborhood. This finding indicates that social support (e.g., seeing many people walking around the neighborhood) and social ties in the neighborhood may promote children’s ATS by decreasing parental safety concerns.

This study has several limitations most of which are common to this type of observational studies carried out in a single community/city setting. First, our study was carried out in Austin, Texas, and therefore its findings are applicable only to those other cities with similar demographic and environmental characteristics. Second, although we selected our study variables based on the previous literature, relevant theories, and data availability, it is likely that there are other variables not considered in this study that are potentially important to understand parental safety concerns. For example, increased parental concerns about safety for children’s ATS may be attributed to their own psychological distress or anxiety. Several studies showed that parents with psychological distress were more likely to perceive low social support and at risk of poor mental health [37,38]. Furthermore, a study shows that children with highly stressed parents were less likely to participate in physical activity than children with normally stressed parents [39]. Thus, future studies need to consider the levels of psychological stress or anxiety of parents when it comes to framing parental concerns for children’s ATS. Such parental responses may also be associated with their perception of the child’s competence or self-efficacy [40] and the child’s use of a mobile phone in ATS.

Third, regarding the natural environmental variables, only three variables maintained their statistical significance in the final model. This is primarily due to the serious multi-collinearity problem for many natural environmental variables, which forced the model to select only a few variables that were mostly independent of each other, not because the other natural environmental variables were not significant. For example, two variables including tree canopy and NDVI (density of green leaves) were highly correlated (r = 0.79), and, thereby, only one variable was used in the NE model although those two variables were significant in one-by-one tests. Lastly, the home-to-school route buffer used for the built and natural environment measurement was based on the shortest home-to-school route, not the actual one. While studies show that most people use the shortest route, it is possible that some use a longer route to avoid unsafe/undesirable areas [41].

Despite these limitations, this study makes an important contribution to the existing literature by employing quantitative and objective methods to better understand how parental perceived barriers to ATS are associated with both the built and the natural environment conditions, which has been rarely examined so far. Future research will be necessary for assessing both the direct and the indirect associations of the environmental conditions with ATS, and for examining whether the parental safety concerns play a mediating role in the environment–ATS relationship.

5. Conclusions

Parental safety concerns (e.g., related to getting lost/bullied/or hurt by a stranger, attacked by stray dogs, hit by a car, exhaust fumes, and eyes on the street) are key determinants influencing parental decision-making related to children’s ATS, and reducing parental concerns is an important a prerequisite for promoting ATS. Given the findings on personal attitudes toward walking and social support, educational/promotional and social participation events in neighborhoods may help reduce parental safety concerns for their children’s ATS. Furthermore, providing walking-friendly built and natural environments (e.g., more sidewalks, absence of highways and railroads, gentle slopes, and wide-canopy trees providing shade along sidewalks) along home-to-school routes may further help lower parental safety concerns for their children’s ATS [42]. These environmental characteristics can be more readily improved than the personal (e.g., socioeconomic status) and policy (e.g., zoning) factors. Therefore, further attention to the need to specifically target parental safety concerns as the most significant yet modifiable barriers to ATS should be considered.

**Author Contributions:** Conceptualization, Y-J.K. and C.L.; methodology, Y-J.K. and C.L.; software, Y-J.K.; validation, Y-J.K. and C.L.; formal analysis, Y-J.K.; investigation, Y-J.K. and C.L; resources, Y-J.K and C.L; data curation, Y-J.K. and C.L.; writing—original draft preparation, Y-J.K.; writing—review and editing, C.L. and Y-J.K.; visualization, Y-J.K.; supervision, C.L. and Y-J.K.; project administration, C.L. and Y-J.K.; Funding Acquisition, C.L. and Y.J.K.

**Funding:** This study was funded by the Robert Wood Johnson Foundation’s Active Living Research program (Grant ID: 65539) and was supported by the 2018 Yeungnam University Research Grant.

**Conflicts of Interest:** The authors declare no conflict of interest.

References

1. U.S. Department of Health and Human Services. Physical Activity Guidelines for Americans. Availabe online: http://www.health.gov/paguidelines/ (accessed on 5 May 2014).

2. Davison, K.K.; Werder, J.L.; Lawson, C.T. Peer reviewed: Children’s active commuting to school: Current knowledge and future directions. *Prev. Chronic Dis.* **2008**, 5, 1–11.

3. Faulkner, G.E.; Buliung, R.N.; Flora, P.K.; Fusco, C. Active school transport, physical activity levels and body weight of children and youth: a systematic review. *Prev. Med.* **2009**, 48, 3–8.

4. Southward, E.F.; Page, A.S.; Wheeler, B.W.; Cooper, A.R. Contribution of the school journey to daily physical activity in children aged 11–12 years. *Am. J. Prev. Med.* **2012**, 43, 201–204.

5. McDonald, N.C.; Brown, A.L.; Marchetti, L.M.; Pedroso, M.S. U.S. School Travel, 2009: An Assessment of Trends. *Am. J. Prev. Med.* **2011**, 41, 146–151.

6. Dalton, M.A.; Longacre, M.R.; Drake, K.M.; Gibson, L.; Adachi-Mejia, A.M.; Swain, K.; Xie, H.; Owens, P.M. Built environment predictors of active travel to school among rural adolescents. *Am. J. Prev. Med.* **2011**, 40, 312–319.

7. Larsen, K.; Gilliland, J.; Hess, P.; Tucker, P.; Irwin, J.; He, M. The Influence of the physical environment and sociodemographic characteristics on children’s mode of travel to and from school. *Am. J. Public Health* **2009**, 99, 520–526.

8. Mitra, R.; Building, R.N.; Roorda, M.J. Built environment and school travel mode choice in Toronto, Canada. *Transp. Res. Rec. J. Transp. Res. Board* **2010**, 2156, 150–159.

9. Panter, J.R.; Jones, A.P.; Van Sluijs, E.M.; Griffin, S.J. Neighborhood, route, and school environments and children's active commuting. *Am. J. Prev. Med.* **2010**, 38, 268–278.

10. Faulkner, G.E.; Richichi, V.; Buliung, R.N.; Fusco, C.; Moola, F. What’s “quickest and easiest?”: Parental decision making about school trip mode. *Int. J. Behav. Nutr. Phys. Act.* **2010**, 7, 1–11.

11. Ahlport, K.N.; Linnan, L.; Vaughn, A.; Evenson, K.R.; Ward, D.S. Barriers to and facilitators of walking and bicycling to school: Formative results from the non-motorized travel study. *Health Educ. Behav.* **2008**, 35, 221–244.

12. Greves, H.M.; Lozano, P.; Liu, L.; Busby, K.; Cole, J.; Johnston, B. Immigrant families' perceptions on walking to school and school breakfast: A focus group study. *Int. J. Behav. Nutr. Phys. Act.* **2007**, 4, 1–9.

13. Panter, J.R.; Jones, A.P.; van Sluijs, E.M.; Griffin, S.J. Attitudes, social support and environmental perceptions as predictors of active commuting behaviour in school children. *J. Epidemiol. Community Health* **2010**, 64, 41–48.

14. Zhu, X.; Lee, C. Correlates of walking to school and implications for public policies: Survey results from parents of elementary school children. *J. Public Health Policy* **2009**, 30, 177–202.

15. Bennetts, S.K.; Cooklin, A.R.; Crawford, S.; D’Esposito, F.; Hackworth, N.J.; Green, J.; Matthews, J.; Strazdins, L.; Zubrick, S.R.; Nicholson, J.M. What influences parents’ fear about children’s independent mobility? Evidence from a state-wide survey of Australian parents. *Am. J. Health Promot.* **2018**, 32, 667–676.

16. Smith, M.; Amann, R.; Cavadino, A.; Raphael, D.; Kearns, R.; Mackett, R.; Mackay, L.; Carroll, P.; Forsyth, E.; Mavoa, S. Children’s transport built environments: a mixed methods study of associations between perceived and objective measures and relationships with parent licence for independent mobility in Auckland, New Zealand. *Int. J. Environ. Res. Public Health* **2019**, 16, 1361.

17. Lee, C.; Kim, Y.-J. Active commuting to school: Personal and environmental factors correlated with parental safety concerns. In Proceedings of Association of European Schools of Planning. Ankara, Turkey, 13 July 2012.

18. Lee, C.; Zhu, X.; Yoon, J.; Varni, J.W. Beyond distance: children's school travel mode choice. *Ann. Behave. Med.* **2013**, 45, S55-S67.

19. Forman, H.; Kerr, J.; Norman, G.J.; Saelens, B.E.; Durant, N.H.; Harris, S.K.; Sallis, J.F. Reliability and validity of destination-specific barriers to walking and cycling for youth. *Prev. Med.* **2008**, 46, 311–316.

20. Hume, C.; Ball, K.; Salmon, J. Development and reliability of a self-report questionnaire to examine children's perceptions of the physical activity environment at home and in the neighbourhood. *Int. J. Behav. Nutr. Phys. Act.* **2006**, 3, 1–6.

21. McMillan, T.E. Walking and Urban Form: Modeling and Testing Parental Decisions about Children’s Travel. Ph.D. Thesis, University of California. Irvine, CA. 2003.

22. Wood, L.; Frank, L.D.; Giles-Corti, B. Sense of community and its relationship with walking and neighborhood design. *Soc. Sci. Med.* **2010**, 70, 1381–1390.

23. Acock, *A Gentle Introduction to Stata*; Stata Press: College Station, TX, USA, 2008.

24. ESRI. Hot Spot Analysis (Getis-Ord Gi*) (Spatial Statistics). Availabe online: http://resources.arcgis.com/en/help/main/10.1/index.html#//005p00000010000000 (accessed on 17 May 2014).

25. Baig, F.; Hameed, M.; Li, M.; Shorthouse, G.; Roalfe, A.; Daley, A. Association between active commuting to school, weight and physical activity status in ethnically diverse adolescents predominately living in deprived communities. *Public Health* **2009**, 123, 39–41.

26. Martin, S.L.; Lee, S.M.; Lowry, R. National prevalence and correlates of walking and bicycling to school. *Am. J. Prev. Med.* **2007**, 33, 98–105.

27. McDonald, N.C. Travel and the social environment: Evidence from Alameda County, California. *Transp. Res. Part D Transp. Environ.* **2007**, 12, 53–63.

28. McDonald, N.C. Critical factors for active transportation to school among low-income and minority students - Evidence from the 2001 national household travel survey. *Am. J. Prev. Med.* **2008**, 34, 341–344.

29. Timperio, A.; Ball, K.; Salmon, J.; Roberts, R.; Giles-Corti, B.; Simmons, D.; Baur, L.A.; Crawford, D. Personal, family, social, and environmental correlates of active commuting to school. *Am. J. Prev. Med.* **2006**, 30, 45–51.

30. StataCorp, L. Stata data analysis and statistical Software. *Spec. Edit. Release* **2007**, 10, 733.

31. Hoehner, C.M.; Brennan Ramirez, L.K.; Elliott, M.B.; Handy, S.L.; Brownson, R.C. Perceived and objective environmental measures and physical activity among urban adults. *Am. J. Prev. Med.* **2005**, 28, 105–116.

32. Ewing, R.; Forinash, C.V.; Schroeer, W. Neighborhood schools and sidewalk connections: What are the impacts on travel mode choice and vehicle emissions? *TR News* **2005**, 4–10.

33. Ewing, R.; Schroeer, W.; Greene, W. School location and student travel analysis of factors affecting mode choice. *Transp. Res. Rec. J. Transp. Res. Board* **2004**, 1895, 55–63.

34. Kerr, J.; Rosenberg, D.; Sallis, J.F.; Saelens, B.E.; Frank, L.D.; Conway, T.L. Active commuting to school: Associations with environment and parental concerns. *Med. Sci. Sports Exerc.* **2006**, 38, 787–794.

35. Mota, J.; Gomes, H.; Almeida, M.; Ribeiro, J.C.; Carvalho, J.; Santos, M.P. Active versus passive transportation to school - differences in screen time, socio-economic position and perceived environmental characteristics in adolescent girls. *Ann. Hum. Biol.* **2007**, 34, 273–282.

36. Schlossberg, M.; Greene, J.; Phillips, P.P.; Johnson, B.; Parker, B. School trips: Effects of urban form and distance on travel mode. *J. Am. Plan. Assoc.* **2006**, 72, 337–346.

37. Al‐Gamal, E.; Long, T. Psychological distress and perceived support among Jordanian parents living with a child with cerebral palsy: A cross‐sectional study. *Scand. J. Caring Sci.* **2013**, 27, 624–631.

38. Hung, J.-W.; Wu, Y.H.; Chiang, Y.-C.; Wu, W.C.; Yeh, C.-H. Mental health of parents having children with physical disabilities. *Chang Gung Med. J.* **2010**, 33, 82–91.

39. Walton, K.; Simpson, J.R.; Darlington, G.; Haines, J. Parenting stress: A cross-sectional analysis of associations with childhood obesity, physical activity, and TV viewing. *BMC Pediatr.* **2014**, 14, 244.

40. Kim, Y.-J.; Lee, C.; Lu, W.; Mendoza, J. Attitudinal, behavioral, and environmental correlates of child and parent self-efficacy in walking to school. *Int. J. Environ. Res. Public Health* **2017**, 14, 1588.

41. Lee, C.; Li, L. Demographic, physical activity, and route characteristics related to school transportation: an exploratory study. *Am. J. Health Promot.* **2014**, 28, S77–S88.

42. Kim, Y.-J.; Lee, C.; Kim, J.-H. Sidewalk landscape structure and thermal conditions for child and adult pedestrians. *Int. J. Environ. Res. Public Health* **2018**, 15, 148.

| 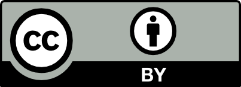 | © 2020 by the authors. Submitted for possible open access publication under the terms and conditions of the Creative Commons Attribution (CC BY) license (http://creativecommons.org/licenses/by/4.0/). |
| --- | --- |
